# Supplementary material for: Symbiotic Cell Differentiation and Cooperative Growth in Multicellular Aggregates
Source: PLoS Comput Biol. 2016 Oct 17;12(10):e1005042. doi: 10.1371/journal.pcbi.1005042 (PMC5066942; doi:10.1371/journal.pcbi.1005042)
Supplement: S1 Text — Figures A and B Typical behavior of categories (a) and (b); Figure C Network 3; Figure D Dependence of the concentration of the nutrient X0 on r(1); Figure E Phase diagrams of the r1cell and r2cell models. (PDF) [file pcbi.1005042.s001.pdf]

Supplemental Material
for "Symbiotic Cell Differentiation and Cooperative Growth in Multicellular Aggregates"

Jumpei Yamagishi,* Nen Saito,† and Kunihiko Kaneko†

1 Details of the N-cell model

Figures A and B show typical behaviors of categories (a) and (b).

For all simulations, we set the number of chemical species to $k = 20$, the number of reaction paths from each chemical as $\rho = 4$, and the probability that a chemical is diffusible as $q = 0.15$. The other parameters are set as $D = D_{med} = 0.1$, $C = 0.05$, and $V_{med} = 100$. D_{med} was set as $D_{med} = D$ for simplicity but, again, the results reported here do not greatly depend on the value of D_{med}. The fourth-order Runge-Kutta method was used to numerically solve Eqs. (1)-(2).

2 Network 3 obtained through the simplification of reaction networks

Network 3, shown in Figure C in S1 Text, exhibits differentiation into two types. In one type, x_1 is high while x_2 is close to zero, and in the other

*College of Arts and Sciences, The University of Tokyo, 4-6-1, Komaba, Meguro-ku, Tokyo 153-8505, Japan

†Graduate School of Arts and Sciences, The University of Tokyo, 3-8-1 Komaba, Meguro-ku, Tokyo 153-8902, Japan

type, x_2 is high and x_1 is close to zero. Moreover, cells in network 3 exhibit balanced, cooperative growth.

3 Note on the $V \to \infty$ limit

It should be noted that when V_{med} or V is sufficiently large, the behavior of interacting cells is equivalent to that of an isolated cell, because a larger V_{med} leads to weaker cell-cell interactions; i.e., the second term of Eq. (2) that is proportional to $1/V_{med}$ can be disregarded. However, this is only the case with small t. Over time, chemicals diffused from N cells gradually accumulate in the medium. Consequently, at an equilibrium state (i.e., for the limit $t \to \infty$), the contribution of the third term is not negligible, so that N cells in a medium can differentiate due to cell-cell interactions, even with a large V_{med}.

4 Explanation of the peaks in Figs 5B and 5C

Note that if V and D_{med} are too small, the amount of nutrient in the medium could be low. Under such a pathological condition, the nutrient is exhausted and thus the influx of the nutrient into the medium from the exterior becomes the rate-limiting step. Hence, R does not monotonously increase as V becomes too small (there is a peak of R_μ; see Fig 5B), although $R_\mu > 1$ is still satisfied. In contrast, when V is not small, the efficiency of nutrient consumption (i.e., the influx of the nutrient into cells from the medium) is the rate-limiting step.

The peak in Fig 5C can be explained by a similar mechanism.

5 Robustness in population distribution

Now, let us consider how the difference in volume alters the states of two interacting cells in networks 1-3. When $r^{(1)} > 1/2$, the amount of X_4 supplied from the minority type-2 cell is not sufficient for the majority type-1 cell to maintain the differentiated chemical composition. Consequently, the state of the type-1 cell approaches that of an isolated cell, so that the cell

de-differentiates to approach the single-cell state. In contrast, the minority type-2 cell receives sufficient amounts of X_3 from the majority type-1 cell, and maintains its differentiated composition. Here, if condition (ii) is satisfied, $x_0^{(iso)} > x_0^{(dif)}$ holds because catalytic reactions and the nutrient consumption are faster in a differentiated cell. Thus, $\partial x_0^{(1)}/\partial r^{(1)} > 0$ holds, as exemplified in Figure D in S1 Text, if de-differentiation occurs monotonously.

The collective growth is, therefore, balanced when the amount of chemicals provided from the majority type exceeds that supplied from the minority type, according to Eq. (3). However, even when the majority type receives a larger amount of total chemicals, the collective growth can be balanced for sufficiently small $\partial x_0^{(1)}/\partial r^{(1)}$; i.e., the majority type refrains from taking the nutrient in, so that $\sum_{i=1}^{k-1} \sigma_i \partial(x_i^{(1)} - x_i^{(2)})/\partial r^{(1)} > -2\partial x_0^{(1)}/\partial r^{(1)}$.

When generalized into the case in which the catalytic network is not symmetric, the dynamics of $r^{(1)}$ are given by

$$\frac{dr^{(1)}}{dt} = r^{(1)}(1 - r^{(1)})[F(r^{(1)}) - G(1 - r^{(1)})]. \tag{S1}$$

When a fixed-point solution is given as $r^{(1)} = r^*$ $(0 < r^* < 1)$, the stability of the fixed point is determined by the sign of $F'(r^*) + G'(1 - r^*)$, which is unstable for $F'(r^*) + G'(1 - r^*) > 0$ and is stable for $F'(r^*) + G'(1 - r^*) < 0$.

6 Linear stability analysis in the r1cell and r2cell models

The phase diagrams, shown in Fig 4A and Figure E in S1 Text, were numerically obtained from the r2cell model. We also performed a linear stability analysis around the homogeneous fixed point, i.e., $x_i^{(1)} = x_i^{(2)}$, for all i.

First, we define the Jacobian matrices that depend on (C, V, D). The

Jacobian matrix of the r1cell and the r2cell models are respectively given by

$$J^{(1)} = \begin{pmatrix} \frac{\partial \dot{x}_0^{(1)}}{\partial x_0^{(1)}} & \cdots & \frac{\partial \dot{x}_0^{(1)}}{\partial x_{k-1}^{(1)}} & \frac{\partial \dot{x}_0^{(1)}}{\partial x_0^{(med)}} & \cdots & \frac{\partial \dot{x}_0^{(1)}}{\partial x_{k-1}^{(med)}} \\ \vdots & \ddots & \vdots & \vdots & & \vdots \\ \frac{\partial \dot{x}_{k-1}^{(1)}}{\partial x_0^{(1)}} & \cdots & \frac{\partial \dot{x}_{k-1}^{(1)}}{\partial x_{k-1}^{(1)}} & \frac{\partial \dot{x}_{k-1}^{(1)}}{\partial x_0^{(med)}} & \cdots & \frac{\partial \dot{x}_{k-1}^{(1)}}{\partial x_{k-1}^{(med)}} \\ \frac{\partial \dot{x}_0^{(med)}}{\partial x_0^{(1)}} & \cdots & \frac{\partial \dot{x}_0^{(med)}}{\partial x_{k-1}^{(1)}} & \frac{\partial \dot{x}_0^{(med)}}{\partial x_0^{(med)}} & \cdots & \frac{\partial \dot{x}_0^{(med)}}{\partial x_{k-1}^{(med)}} \\ \vdots & & \vdots & \vdots & \ddots & \vdots \\ \frac{\partial \dot{x}_{k-1}^{(med)}}{\partial x_0^{(1)}} & \cdots & \frac{\partial \dot{x}_{k-1}^{(med)}}{\partial x_{k-1}^{(1)}} & \frac{\partial \dot{x}_{k-1}^{(med)}}{\partial x_0^{(med)}} & \cdots & \frac{\partial \dot{x}_{k-1}^{(med)}}{\partial x_{k-1}^{(med)}} \end{pmatrix},$$

$$J^{(2)} = \begin{pmatrix} \frac{\partial \dot{x}_0^{(1)}}{\partial x_0^{(1)}} & \cdots & \frac{\partial \dot{x}_0^{(1)}}{\partial x_{k-1}^{(2)}} & \frac{\partial \dot{x}_0^{(1)}}{\partial x_0^{(med)}} & \cdots & \frac{\partial \dot{x}_0^{(1)}}{\partial x_{k-1}^{(med)}} \\ \vdots & \ddots & \vdots & \vdots & & \vdots \\ \frac{\partial \dot{x}_{k-1}^{(2)}}{\partial x_0^{(1)}} & \cdots & \frac{\partial \dot{x}_{k-1}^{(2)}}{\partial x_{k-1}^{(2)}} & \frac{\partial \dot{x}_{k-1}^{(2)}}{\partial x_0^{(med)}} & \cdots & \frac{\partial \dot{x}_{k-1}^{(2)}}{\partial x_{k-1}^{(med)}} \\ \frac{\partial \dot{x}_0^{(med)}}{\partial x_0^{(1)}} & \cdots & \frac{\partial \dot{x}_0^{(med)}}{\partial x_{k-1}^{(2)}} & \frac{\partial \dot{x}_0^{(med)}}{\partial x_0^{(med)}} & \cdots & \frac{\partial \dot{x}_0^{(med)}}{\partial x_{k-1}^{(med)}} \\ \vdots & & \vdots & \vdots & \ddots & \vdots \\ \frac{\partial \dot{x}_{k-1}^{(med)}}{\partial x_0^{(1)}} & \cdots & \frac{\partial \dot{x}_{k-1}^{(med)}}{\partial x_{k-1}^{(2)}} & \frac{\partial \dot{x}_{k-1}^{(med)}}{\partial x_0^{(med)}} & \cdots & \frac{\partial \dot{x}_{k-1}^{(med)}}{\partial x_{k-1}^{(med)}} \end{pmatrix},$$

where $\dot{x}_i^{(m)}$ is the right-hand side of Eq. (1) and $\dot{x}_i^{(med)}$ is the right-hand side of Eq. (2).

Next, we define $\lambda_{max}^{(1)}$ as the eigenvalue of $J^{(1)}$ with the largest real part, and also denote the largest eigenvalue of $J^{(2)}$ among the real eigenvalues by $\lambda_R^{(2)}$, and the eigenvalue of $J^{(2)}$ with the largest real part among complex eigenvalues with nonzero imaginary parts by $\lambda_C^{(2)}$. The phase diagrams can be explained with $\lambda_{max}^{(1)}$, $\lambda_R^{(2)}$ and $\lambda_C^{(2)}$.

When $\lambda_{max}^{(1)} > 0$, the chemical concentrations in the r1cell model oscillate, whereas they are stationary when $\lambda_{max}^{(1)}$ is negative. Here, $Re(\lambda_{max}^{(1)}) = 0$ is fulfilled along the red lines in Fig 4A and Figure E in S1 Text.

With cell-cell interaction in the r2cell model, the homogeneous state is unstable for $\lambda_R^{(2)} > 0$ or $Re(\lambda_C^{(2)}) > 0$. Hence, $\lambda_R^{(2)} < 0$ and $Re(\lambda_C^{(2)}) < 0$ are satisfied in phase (I), and the boundary is determined from these conditions. The red line is given as $Re(\lambda_C^{(2)}) = 0$, in which $Re(\lambda_{max}^{(1)}) = 0$ is also satisfied,

while the green line is determined by $\lambda_R^{(2)} = 0$. Each λ is positive to the left of each line.

The boundary of phases (II) and (III) is given by $\lambda_C^{(2)} = 0$ and $\lambda_R^{(2)} > 0$. This is because when $\lambda_R^{(2)} > 0$ and $Re(\lambda_C^{(2)}) < 0$, pitchfork-type differentiation occurs regardless of the initial state, while oscillation-death differentiation or synchronized oscillation occurs if $\lambda_R^{(2)} > 0$ and $Re(\lambda_C^{(2)}) > 0$.

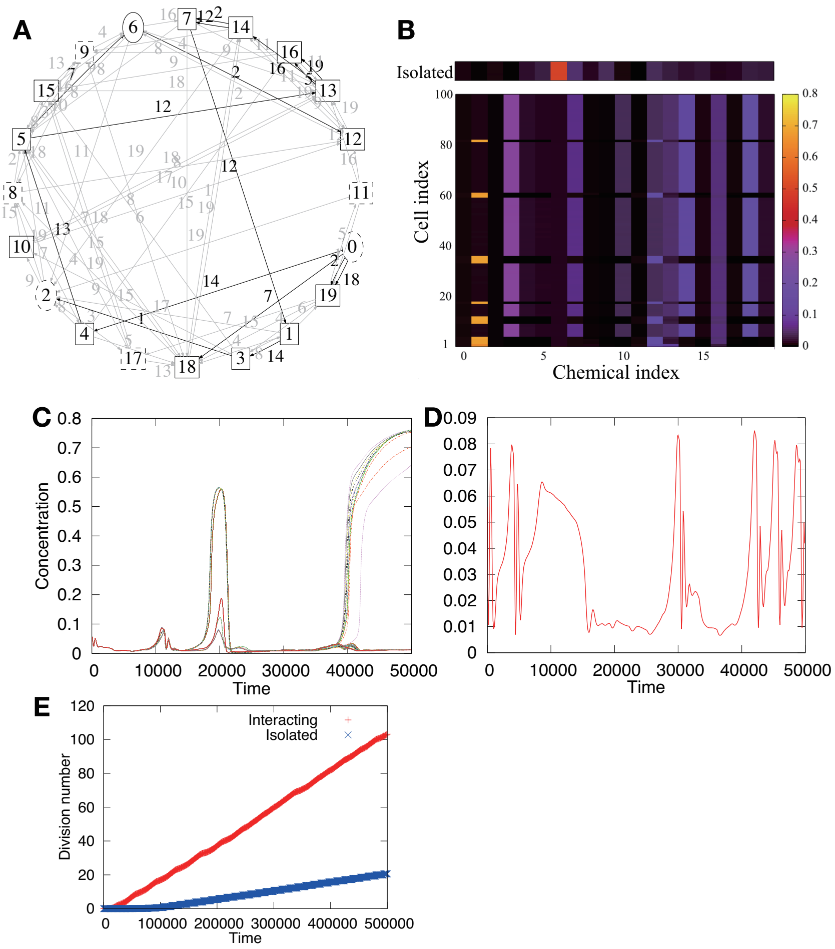

Figure A: Typical behavior of category (a): Interacting cells can exhibit differentiation, and differentiated cells grow faster than isolated cells ($R_\mu > 1$). (A) An example of catalytic reaction networks. (B) Chemical compositions of N cells surviving at time $t = 5\times10^5$. The concentration $x_i^{(m)}$ is plotted with a color code, with the vertical axis as the cell index m, and the horizontal axis as the chemical index i; the top band designates the composition of an "isolated" cell. Cells differentiate into two types with distinct compositions. (C) The time series of $x_1^{(m)}$ in interacting N cells surviving at time $t = 5\times10^4$, overlaid for all cells shown in different colors. (D) The time series of the concentration of X_1 in the isolated cell. (E) The time series of the number of cell divisions per interacting (red) and isolated cell. In (C) and (D), different colors represent the concentrations of different cells, and the time series of concentrations of surviving N cells at time $t = 5\times10^4$ are shown.

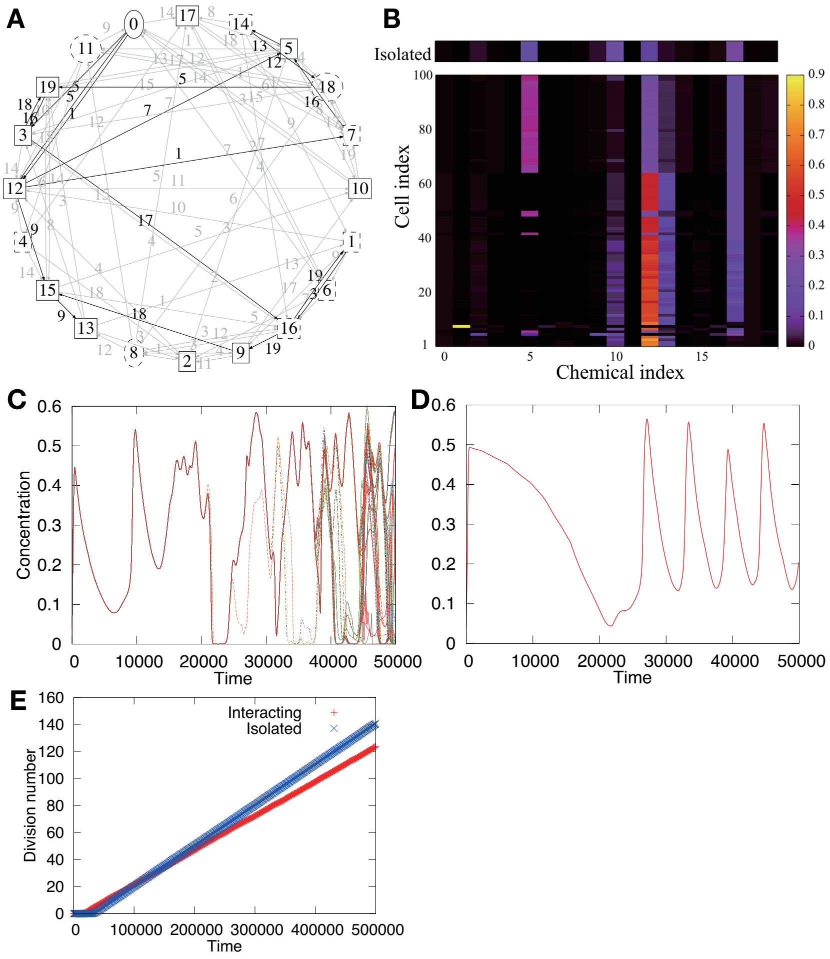

Figure B: Typical behavior of category (b): Interacting cells can exhibit differentiation but differentiated cells grow slower than isolated cells ($R_\mu < 1$). (A) An example of a catalytic reaction network. (B) Chemical compositions of N cells surviving at time $t = 5{\times}10^5$. The concentration $x_i^{(m)}$ is plotted with a color code, with the vertical axis as the cell index m and the horizontal axis as the chemical index i; the top band designates the composition of an "isolated" cell. Cells differentiate into two types with distinct compositions. (C) The time series of $x_5^{(m)}$ in interacting N cells surviving at time $t = 5{\times}10^4$, overlaid for all cells with different colors. (D) The time series of the concentration of X_5 in the isolated cell. (E) The time series of the number of cell divisions per interacting (red) and isolated cell. In (C) and (D), different colors signify the concentrations of different cells, and the time series of concentrations of surviving N cells at time $t = 5{\times}10^4$ are shown.

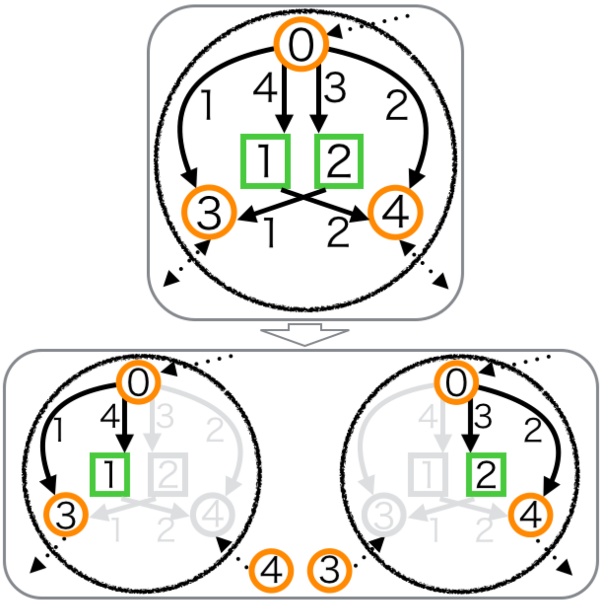

Figure C: Network 3. Subnetworks in the lower panel are active when the cells differentiate, and the chemical at the arrowhead is transformed to the chemical at the arrowtail, catalyzed by the chemical labeled on the edge. Orange circled and green square nodes respectively signify diffusible and non-diffusible chemicals.

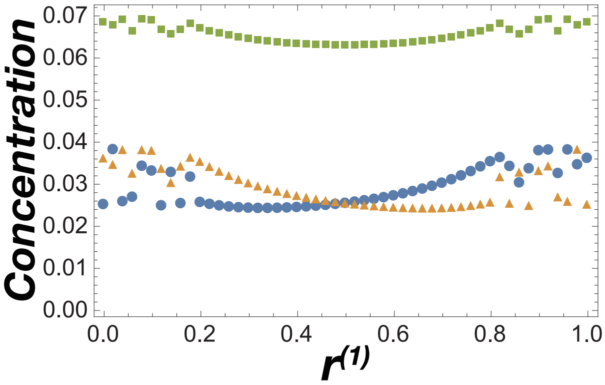

Figure D: Dependence of the concentration of X_0 in network 1 on $r^{(1)}$ ($D = D_{med} = 0.1$). Blue circles, orange triangles, and green rectangles represent the concentration in type-1 $x_0^{(1)}$, type-2 $x_0^{(2)}$, and the medium $x_0^{(med)}$, respectively. At $r^{(1)} = 1/2$, the gradient of the blue line is positive, i.e., $\partial x_0^{(1)}/\partial r^{(1)} > 0$.

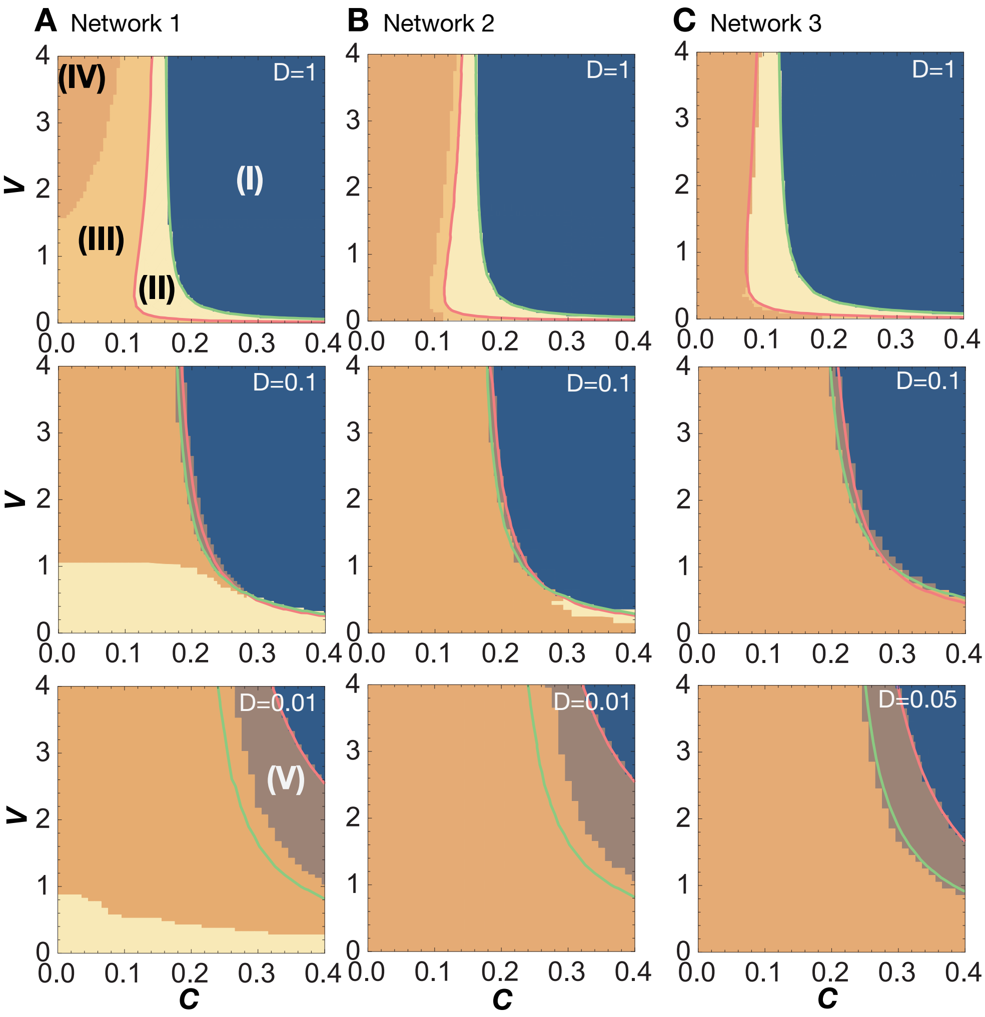

Figure E: Phase diagrams of the r1cell and r2cell models. (A) Network 1. (B) Network 2. (C) Network 3. The blue area designates phase (I): Cells cannot exhibit differentiation, and go to a single fixed point in both the r1cell and r2cell models. In phases (II) and (III) that are separated by the red line, cells always differentiate. In phase (II), as shown by the cream color, "pitchfork-type" differentiation occurs as shown in B, while in phase (III), as shown by the light-orange-color, "oscillation-death" bifurcation occurs as shown in C. In phase (IV), shown in orange, cells in the r2cell model exhibit "oscillation-death" differentiation or synchronized oscillation, depending on the initial cellular state. In phase (V), shown in brown, cells always exhibit synchronized oscillation. Hence, cells in the r2cell model cannot differentiate in phases (I) and (V).
